# Whole Genome Sequencing in an Acrodermatitis Enteropathica Family from the Middle East

**DOI:** 10.1155/2018/1284568

**Published:** 2018-08-07

**Authors:** Faisel Abu-Duhier, Vivetha Pooranachandran, Andrew J. G. McDonagh, Andrew G. Messenger, Johnathan Cooper-Knock, Youssef Bakri, Paul R. Heath, Rachid Tazi-Ahnini

**Affiliations:** ^1^Prince Fahd Bin Sultan Research Chair, Department of Medical Lab Technology, Faculty of Applied Medical Science, Prince Fahd Research Chair, University of Tabuk, Tabuk, Saudi Arabia; ^2^Department of Neuroscience, SITraN, The Medical School, University of Sheffield, Sheffield S10 2RX, UK; ^3^Department of Dermatology, Royal Hallamshire Hospital, Sheffield S10 2JF, UK; ^4^Department of Infection, Immunity and Cardiovascular Disease, The Medical School, University of Sheffield, Sheffield S10 2RX, UK; ^5^Biology Department, Faculty of Science, University Mohammed V Rabat, Rabat, Morocco; ^6^Laboratory of Medical Biotechnology (MedBiotech), Rabat Medical School and Pharmacy, University Mohammed V Rabat, Rabat, Morocco

## Abstract

We report a family from Tabuk, Saudi Arabia, previously screened for Acrodermatitis Enteropathica (AE), in which two siblings presented with typical features of acral dermatitis and a pustular eruption but differing severity. Affected members of our family carry a rare genetic variant, p.Gly512Trp in the SLC39A4 gene which encodes a zinc transporter; disease is thought to result from zinc deficiency. Similar mutations have been reported previously; however, the variable severity within cases carrying the p.Gly512Trp variant and in AE overall led us to hypothesise that additional genetic modifiers may be contributing to the disease phenotype. Therefore whole genome sequencing was carried out in five family members, for whom material was available to search for additional modifiers of AE; this included one individual with clinically diagnosed AE. We confirmed that the p.Gly512Trp change in SLC39A4 was the only candidate homozygous change which was sufficiently rare (ExAC allele frequency 1.178e-05) and predicted deleterious (CADD score 35) to be attributable as a fully penetrant cause of AE. To identify other genes which may carry relevant genetic variation, we reviewed the relevant literature and databases including Gene Ontology Consortium, GeneMANIA, GeneCards, and MalaCards to identify zinc transporter genes and possible interacting partners. The affected individual carried variants in RECQL4 and GPAA1 genes with ExAC allele frequency <0.01 and CADD score >10. p.Gly512Trp is highly likely to be the pathogenic variant in this family. This variant was previously detected in a Tunisian proband with perfect genotype-phenotype segregation suggestive of pathogenicity. Further research is required in this area due to small sample size, but attention should be given to* RECQL4* and* GPAA1* to understand their role in the skin disease.

## 1. Introduction

Acrodermatitis Enteropathica (AE) is a rare inherited metabolic condition that affects zinc absorption and inheritance is often seen in an autosomal recessive pattern [1]. The frequency of inherited cases is estimated at 1:500,000 individuals with no obvious correlation in race or gender [2]. The manifestations of AE include alopecia, diarrhoea, dermatitis, growth retardation, and behavioural changes. Although AE has been attributed to fully penetrant homozygous mutations in the SLC39A4 gene, severity is variable even within individual families [3] suggesting that additional genetic modifiers may play a role.

Measurement of plasma zinc is the commonest method of identifying patients with AE, and reduced levels <60 ug/dL were noted in patients B03 and B06 in the original study which were correlated with their typical AE symptoms. However, limitations of zinc testing have been highlighted by Garza-Rodriguez et al. (2015) [4] who reported a rare case of AE presenting with two novel missense mutations of the SLC39A4 gene associated with periorificial and acral dermatitis only and with normal plasma levels of zinc.

**Figure 1:**
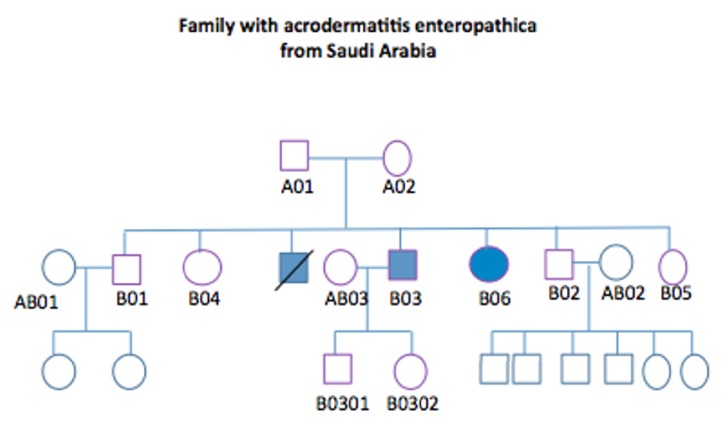
Pedigree analysis to demonstrate the family of AE and the sequenced individuals for this study. Genome sequence of the following samples: A01, A02, B03, AB01, and AB02. Only B03 and B06 are affected (one male sibling was also affected, but he died). Note. Shading in blue displays the individuals affected by the disease.

The family in this study was previously screened and analysed by Abu-Duhier et al. 2017 [3] to identify the pathogenic mutation. In this analysis DNA samples obtained from eleven individuals were amplified by polymerase chain reaction and Sanger sequenced. This comprised both parents and all the siblings including the affected individuals with their respective partners. A homozygous alternate allele in chromosome 8 at position 145638714 was identified in the gene SLC39A4 (solute carrier 39 member 4) which is a zinc transporter. This mutation was present only in the two affected members B03 and B06 presenting with the AE phenotype (Figure 1). The mutation gave rise to a change from the neutral aliphatic amino acid glycine to the highly hydrophobic aromatic tryptophan, with genotype c.1534G>T at position p.Gly512Trp. Although there was strong evidence that this mutation is the cause of AE in this family, severity was variable suggesting that other modifiers may be present. Moreover, because sequencing was limited to a small number of genes, it is possible that alternative homozygous pathogenic change in a zinc transporter gene may have been missed. Others have reported the p.Gly512Trp change in a patient with AE [1], but the role of genetic modifiers has not previously been examined.

To exhaustively define the genetic basis of AE in our patients, the whole genome of available family members has now been evaluated. In this study, we aimed to identify pathogenic and relevant modifier genes associated with familial AE. On this occasion the proband B03, his parents A01 and A02, and unaffected family members AB01 and AB02 were examined by whole genome sequencing. Two additional family members were utilised as population controls in order to eliminate shared, but nonrelevant alleles.

## 2. Materials and Methods

Ethical approval was obtained from both University of Tabuk and University of Sheffield, and written informed consent was obtained to use genetic data for research purposes. Blood samples were available from 5 members of the family with hereditary AE and were initially sequenced (as discussed above), followed by whole genome sequencing. The individuals studied were the affected child B03, his parents A01 and A02, and two unrelated family members AB01 and AB02 who were included as individuals from the same population background (Figure 1). Both lanes of three HiSeq 2500 Rapid Run were used to multiplex and sequence the five samples. To produce fastq files, bcl2fastq version 1.8.4 was used with adapter trimming further modified to accept a single mismatch in the index sequence. BWA ALN version 0.7.5a was used to map reads by lane to the human reference genome hg19. Picard version 1.101 was used to mark duplicate reads followed by realignment around InDels using GATK version 2.6-5-gba531bd. Picard was then used to merge lane-level bam files, with additional marking of duplicates and realignment around InDels on the complete bam file. GATK HaplotypeCaller was used to call variants. The minimal calling quality was accepted at 1, allowing 10 alternative haplotypes. GATK was further used to calculate coverage statistics.

Variant calling files of the five individuals were automatically annotated using wANNOVAR to provide information including genomic annotation, frequency of variant observed in controls, and CADD scores. “R” software was used to analyse the annotated variants. Relevant literature was reviewed and GO consortium data used to identify all zinc transporter genes including SLC39A4. Variants in exonic regions of zinc transport genes were filtered by ExAC frequency and CADD score using “R” to identify rare, predicted deleterious variants present in a homozygous form (see sup materials). An ExAC frequency <0.01 and CADD score >10 were employed to suggest a deleterious variant. (ExAC allele frequency suggests the relative frequency of an allele at a genetic locus in a population [5]; CADD score helps measure the level of toxicity of a genetic variant such that a value of 10 defines the variant to be within 10% of a damaging variant in the human genome.) The analysis was repeated in the other sequenced individuals to determine whether the toxic mutation was present in relatives.

To extend the analysis beyond zinc transporter genes, GeneMANIA was used to identify genetic interactions of SLC39A4. Interacting partners were screened for potential rare deleterious variants although the requirement for homozygosity was relaxed to include heterozygous variants.

## 3. Results

### 3.1. Only the Homozygous p.Gly512Trp SLC39A4 Variant Is Sufficiently Rare and Deleterious to Cause AE in Our Pedigree

The identified deleterious SLC39A4 variant p.Gly512Trp (with ExAC frequency of 1.178e-05 and CADD score of 35) was present in homozygous form in B03 (Table 1). As would be expected the same p.Gly512Trp change was found to be present in an heterozygous form in both parents, A01 and A02, but was not found in the unaffected relatives. The remainder of identified variants in SLC39A4 and all other screened zinc transport genes demonstrated either a low CADD score or a high ExAC frequency implying a benign or uncertain significance (Sup Materials 2).

### 3.2. Screening SLC39A4 Interacting Partners Identified Rare Deleterious Variants in Candidate Modifier Genes

The patient B03 suffered a particularly severe form of AE and therefore is a good candidate to identify additional deleterious genetic modifiers of AE. To identify candidate genes which are known to interact with SLC39A4, we used GeneMANIA (https://genemania.org) to identify relevant protein-protein interactions, coexpressed genes, and genes of related function based on transcription and phenotypic screening profiles. The interaction databases used by GeneMANIA included BioGRID and Pathway Commons, both including primary research studies. Databases GeneCards and MalaCards were then used to obtain information on the identified genes (Sup Materials 3). The interacting genes were screened for rare deleterious variants which may have negatively impacted upon the function of SLC39A4 and thus the AE phenotype in our patient. With specific focus on exonic regions of the genes and nonsynonymous mutations, “R” analysis demonstrated two genes that were within acceptable ExAC allele frequency and CADD score range (<0.01, >10): GPAA1 and RECQL4, suggestive of significant interaction with SLC39A4 in developing the disease phenotype in B03.

## 4. Discussion

AE appears to occur mainly in France and Tunisia. The p.Gly512Trp variant was previously identified in a Tunisian family [1]. All published mutations in the human SLC39A4 gene have been collated (Table 2). In the majority of cases, pathogenic variants were present in a homozygous or compound heterozygous state in AE. Families identified across a number of countries with AE have been shown to carry over 30 different mutations in SLC39A4, including deletions, nonsense, missense, and splice-site alterations [6]. Schmitt et al. [1] reported mutations including p.Gly512Trp and p.549delLeu affecting amino acids that were highly conserved between a number of species. Variants p.Gly512Trp and p.549delLeu were detected in homozygous state in Tunisian and Swedish probands, respectively, and the toxic mutation was not identified in any of 164 control chromosomes from North Africa included in their study. Moreover, there was perfect segregation between the genotype and phenotype in the pedigree suggesting a high likelihood for AE to be associated with SLC39A4 gene. As previously described by Abu-Duhier et al. [3], the mutation was located in a putative transmembrane domain and likely to alter zinc absorption by reducing transcription/ translation of SLC39A4. Heterozygous individuals tend to be asymptomatic carriers rather than manifesting the disease phenotype [7]. A significant feature of AE overall and our family in particular is variable phenotype even between individuals with the same genetic change in SLC39A4 [3]. It is anticipated the AE phenotype could be dependent on either modifier genes or an unknown putative AE gene. Hence we have screened for additional genetic modifiers of SLC39A4 function within an individual with a particularly severe AE phenotype [3].

Our data suggests that the p.Gly512Trp change is the likely cause of disease in our family because it is the only homozygous, rare, and predicted deleterious change within a zinc transporter gene which is present within the affected patient and absent (or heterozygous) in unaffected family members. In addition we identified two rare and predicted deleterious genetic variants in SLC39A4 interacting partners which may have a role in the development of the particularly severe AE phenotype in our patient: RECQL4 and GPAA1 (Table 4). Interestingly Nistor et al. [8] reported that the complete clinical triad of features in AE was only documented in 20% of patients, questioning if only one gene is responsible and if modifier genes could be involved in the variable clinical characteristics. Patients with mutation in the RECQL4 gene have several characteristic features similar to AE; for example, Bernstein et al. [9] reported a RECQ disorder: Bloom syndrome, a rare autosomal recessive condition, which presents with mental retardation, immunodeficiency, male infertility, and increased chance of cancer. Similar features occur in some patients with AE, in the form of immunodeficiency, mental retardation, and infertility. In addition, Mann et al. [10] identified distinctive skin abnormalities in a mutant RECQL4 mouse model. Although the GPAA1 gene is known to be expressed in skin (Table 3), no disorder has been identified to date that has been directly affected by GPAA1 genetic mutation, although rare cases have been reported in which GPAA1 gene amplification and RNA and protein overexpression occurred in hepatocellular carcinoma [11]. Further research is required to explore a putative link between GPAA1 and RECQL4 mutations and AE.

Our data supports pathogenicity of the p.Gly512Trp change in SLC39A4, but in addition we have identified potentially deleterious variants in SLC39A4 interacting partners which may be important to disease pathogenesis and are potential therapeutic targets.

## Figures and Tables

**Table 1:** “R” software was used to identify the deleterious zinc transporter gene in B03. An EXAC frequency of <0.01 and CADD score of >10 were accepted. Excluding synonymous variants, at position Chr8:145638714 of the SLC39A4 gene, c.G1534T and p.G512W, portrayed as a rare mutation in comparison to other identified genes and genetic variants. Note. Please see Sup Materials 2 for complete list of zinc transporter genes derived by “R” software for B03.

**Gene**	**Chromosome**	**Start-End**	**Exon**	**Nucleotide substitution**	**Protein substitution**	**EXAC Frequency**	**CADD Score**
SLC39A4	Chr8	145638714	10	c.G1534T	p.G512W	1.178e-05	35

**Table 2:** Modified table from Schmitt et al. (2009) on genetic mutations identified up to date with AE associated SLC39A4 gene. Outlined in bold is the mutation of interest that has been identified in a Tunisian family. Note. Some reports failed to provide adequate information to complete the table.

**Exon**	**Mutation (Nucleotide)**	**Influence on Amino Acid**	**Clinical Significance**	**Other findings**	**Study**
Exon 6	c.1191insC	p.Gln398fsX18	Pathogenic	(i) Homozygous (ii) Frameshift	Coromilas et al. 2011

Exon 5	c.850G>A	p.Glu284Lys	Likely/benign	(i) Missense (ii) France (iii) Homozygous	Kury et al., 2002

Exon 1	c.184T>C	p.Cys62Arg	Uncertain Significance	(i) Missense (ii) May affect protein conformation due to loss of disulfide bond (iii) Tunisia (iv) Homozygous	Kury, et al. 2003

Exon 1	c.143T>G	p.Leu48X	Likely pathogenic	(i) Nonsense (ii) Homozygous (iii) Tunisia (iv) Lacks putative zinc binding site	Kury et al. 2003 Kharfi et al. 2005 Nakano et al. 2003

Intron 1	c.192+19G>A	Donor splice site error (possibly)	Likely /Pathogenic	(i) France (ii) Compound heterozygous/homozygous (iii) Possibly altering transcripts through mis-splicing	Kury et al. 2002

Exon 2	c.283C>T	p.Arg95Cys	Pathogenic	(i) Missense (ii) Japanese (iii) Compound heterozygous (iv) Abolishes restriction enzyme site for Faul	Nakano et al. 2003

Exon 2	c.318C>A	p.Asn106Lys	Pathogenic	(i) Missense (ii) France (iii) Compound Heterozygous (iv) Deletion in one allele – failed expression of gene	Wang et al. 2002

Intron 2	c.475-2A>G	Acceptor splice site error (possibly)	Uncertain Significance	(i) Nonsense (ii) Homozygous (iii) France (iv) Appearance of premature stop codon	Kury et al. 2003

Exon 2	c.251C>T	p.Pro84Leu	Likely Benign	(i) Missense (ii) Various countries (iii) Possibly compound heterozygous (iv) Various amino acid change	Wang et al. 2002

Exon 3	c.511G>T	p.Val171Leu	Uncertain Significance	(i) Missense (ii) Heterozygous (iii) Caucasian	Schmitt et al. 2009

Exon 3	c.599C>T	p.Pro200Leu	Pathogenic	(i) Missense (ii) reduces Vmax/alter protein folding (iii) France + Austria (iv) Compound heterozygous/homozygous	Kury et al. 2002

Exon 3	c.631C>T	p.Gln211X	Likely Pathogenic	(i) Nonsense (ii) Truncated protein (iii) Tunisia (iv) Homozygous	Meftah et al. 2006

Exon 3	c.641_642ins10	p.Ser214ArgfsX30	Unknown	(i) Frameshift	Santiago et al. 2011

Exon 4	c.766delC	p.Leu256SerfsX16	Likely Pathogenic	(i) Deletion (ii) Spanish (iii) Truncated protein (iv) Heterozygous	Schmitt et al. 2009

Exon 4	c.751C>T	p.Arg251Trp	Benign	(i) Missense (ii) Homozygous (iii) France	Wang et al. 2002

Exon 5	c.850G>A	p.Glu284Lys	Likely/benign	(i) Missense (ii) Homozygous	Kury et al. 2003

Exon 5	c.909G>C	p.Gln303His	Pathogenic	(i) Missense (ii) Homozygous (iii) Substitution of highly conserved amino acid (iv) Japan	Nakano et al. 2003

Exon 5	c.926G>A	p.Cys309Tyr	Unknown	(i) Missense	Wang et al. 2002

Exon 5	c.968_971del AGTC	p.Ser324ArgfsX24	Likely/ Pathogenic	(i) Frameshift (ii) France (iii) Compound Heterozygous (iv) Alter protein function	Kury et al. 2002

Exon 6	c.989G>A	p.Gly330Asp	Likely Pathogenic	(i) Cellular mislocalization (ii) Missense (iii) Egypt (iv) Homozygous	Wang et al. 2002

Exon 6	c.1016_1017ins53	p.Thr357AlafsX10	Unknown	(i) Frameshift (ii) Premature termination codon (iii) Heterozygous (iv) Japan	Nakano et al. 2003

Exon 6	c.1115T>C	p.Leu372Pro	Likely Pathogenic	(i) Reduced protein levels (ii) Missense (iv) Egypt (iv) Homozygous	Wang et al. 2002

Exon 6	c.1120G>A	p.Gly374Arg	Pathogenic	(i) Reduced protein levels (ii) Missense (iii) France (iv) Homozygous	Kury et al. 2002

Exon 6	c.1141A>G	p.Thr381Ala	Uncertain Significance	(i) Missense (ii) Heterozygous (iii) Caucasian	Schmitt et al. 2009

Exon 6	c.1115T>G	p.Leu372Arg	Unknown	(i) Missense	Li et al. 2010

Intron 6	c.1150-2A>G	Acceptor splice site error (possibly)	Uncertain significance	(i) Homozygous (ii) France	Wang et al. 2002

Exon 7	c.1203G>A	p.Trp401X	Likely/ pathogenic	(i) Nonsense (ii) Compound Heterozygote (iii) Austria (iv) Absence of zinc binding site	Kury et al. 2003

Exon 7	c.1223delC	p.Ala408fsX481	Unknown	(i) Frameshift	Vardi et al. 2009

Exon 7	c.1223_1227delCCGGG	p.Trp411ArgfsX7	Uncertain significance	(i) Frameshift (ii) Founder Effect (iii) Tunisian (iv) Homozygous	Kury et al. 2002

Exon 7	c.1229T>C	p.Leu410Pro	Uncertain Significance	(i) Missense	Wang et al. 2002

Intron 7	c.1287+2T>C	Acceptor splice site error (possibly)	Uncertain Significance		Park et al. 2010

Exon 9	c.1438G>T	p.Glu480Sto	Unknown	Stop	Nakano et al. 2009

Exon 9	c.1462_147411+delAGACTGAGCCCAGG	p.Arg488SerfsX2	Unknown	(i) Frameshift	Wang et al. 2008

**Exon 10**	**c.1534G>T**	**p.Gly512Trp**	**Pathogenic**	**(i) Missense** **(ii) Tunisia** **(iii) Homozygous** **(iv) Affect amino acids**	**Schmitt et al. 2009**

Exon 10	c.1576G>A	p.Gly526Arg	Pathogenic	(i) Reduces Vmax (ii) Missense (iii) France (iv) Homozygous	Kury et al. 2002

Exon 11	c.1784G>T	p.Gly595Val	Uncertain significance	(i) Missense (ii) Tunisia	Kharfi et al. 2010

Exon 11	c.1646_1648delTGC	p.549delLeu	Pathogenic	(i) Deletion (ii) Sweden (iii) Homozygous (iv) Affect amino acids	Schmitt et al. 2009

Exon 12	c.1888G>C	p.Gly630Arg	Pathogenic for mental retardation/X-Linked	(i) Reduced protein levels (ii) Missense (iii) Homozygous (iv) Jordan	Wang et al. 2002

**Table 3:** GeneMANIA presented the interacting partners with SLC39A4 gene. The databases GeneCards and MalaCards and the literature were reviewed to identify the function, disorder, and tissue expression. Note. The documented information only includes the main signs, symptoms, and tissue expression.

**Gene**	**Description**	**Disease**	**Function**	**Signs/Symptoms**	**Tissue Expression**
SLC39A4	Solute Carrier Family 39 Member 4	Acrodermatitis enteropathica	Encodes for a ZIP family. Required for zinc uptake in the intestine.	Growth retardation, immune-system dysfunction, alopecia, diarrhea, dermatitis.	Mostly Lungs and intestine. Overexpressed in small intestine, fetal gut and CD8 Tcells.

TSTA3	Tissue Specific Transplantation Antigen P35B	Leukocyte adhesion deficiency, type II	Catalyzes the reactions epimerase and reductase in GDP-D-mannose.	Infections, persistent leukocytosis, mental and growth retardation.	Esophagus, stomach and pancreas. Overexpressed in oral epithelium and breast

SCRIB	Scribbled Planar Cell Polarity Protein	Neural tube defects. Tick-Borne Encephalitis.	Regulates epithelial and neuronal morphogenesis.	Cleft lip, myelocystocele, urinary incontinence, hydrocephalus and lipomas.	Intestine, Ovary and Testis. Overexpressed in pancreas.

GPAA1	Glycosylphosphatidylinositol Anchor Attachment	N/A	Links proteins to cell surface membrane.	N/A	Nervous system, skin, lungs. Overexpressed in Nasal epithelium.

CYC1	Cytochrome C1	Mitochondrial complex III deficiency	Mediates the transfer of electron from Rieske iron sulphur protein to cytochrome.	Lactic acidosis, infection, insulin-responsive hyperglycemia and ketoacidosis.	Lungs, Skin, Nervous system. Overexpressed in heart.

RECQL4	RecQ Like Helicase 4	Baller-Gerold Syndrome. RAPADILINO Syndrome.	DNA helicases unwind double stranded DNAs and may modulate chromosome segregation.	Fusion of bones (neonates), slow growth, missing/malformed kneecaps.	Ubiquitously expressed. Overexpressed in Testis and Thymus.

EXOSC4	Exosome Component 4	N/A	Participates in cellular RNA processing and degradation.	N/A	Skin. Overexpressed in whole blood, testis and breast.

**Table 4:** GPAA1 and RECQL4 genes both demonstrated a likelihood of being modifier genes of SLC39A4 in developing AE in B03. Note. Please see Sup Materials 3 for “R” software version of complete list of interacting partners with SLC39A4 gene for B03.

**Gene**	**Chromosome**	**Start-End**	**Exon**	**Nucleotide substitution**	**Protein substitution**	**EXAC Frequency**	**CADD Score**
GPAA1	Chr8	145140564	11	c.G1540A	p.A514T	0.0053	15.04
RECQL4	Chr8	145740364	9	c.C1576T	p.L526F	0.0007	16.37

## Data Availability

The data used to support the findings of this study are available from the corresponding author upon request.

## Supplementary Materials

Supplementary Materials Supplementary materials include codes derived for “R” software analysis of the genome data, code used to identify the mutated zinc transporter genes in B03 sample, and code used to identify interacting partners with SLC39A4 gene in causing the disease in B03.
