# Supplementary material for: Whole Genome Sequencing in an Acrodermatitis Enteropathica Family from the Middle East
Source: Dermatol Res Pract. 2018 Aug 7;2018:1284568. doi: 10.1155/2018/1284568 (PMC6106946; doi:10.1155/2018/1284568)
Supplement: Supplementary Materials — include codes derived for “R” software analysis of the genome data, code used to identify the mutated zinc transporter genes in B03 sample, and code used to identify interacting partners with SLC39A4 gene in causing the disease in B03. [file 1284568.f1.docx]

**Sup materials 1**

**Codes derived for ‘R’ software analysis of the genome data. This language is to include the zinc transporter genes identified by GO Consortium, mutation scoring databases and annotated VCF files of AB02, AB01, A01, A02 and B03 in order to identify the mutation. Specific focus was on homozygous mutations.**

library (data.table)

setwd("C:/Users/sann1458/Documents/Saudi.WGS/txtannovar")

load ("150617.RData")

#AB02 <- fread("9AB02.hg19_multianno.txt",sep="\t",fill=TRUE)

#A02 <- fread("6A02.hg19_multianno.txt",sep="\t",fill=TRUE)

#AB01 <- fread("4AB01.hg19_multianno.txt",sep="\t",fill=TRUE)

#B03 <- fread("13B03.hg19_multianno.txt",sep="\t",fill=TRUE)

#A01 <- fread("myanno.hg19_multianno.txt",sep="\t",fill=TRUE)

#####affected pt

B03.exonic <- (B03[,6])

B03.exonic <- B03.exonic[[1]]

B03.exonic <- which (B03.exonic %in% "exonic")

B03.exonic <- B03[c(B03.exonic),]

zn <- read.table ("ZincTransport.Genes.txt", header=F)

zn <- zn[,1]

read <- B03.exonic[,67]

read <- read[[1]]

hom <- rep(1,length (read))

read <- strsplit(read,":")

for (i in 1:length (hom))

{

a <- read[[i]]

j <- length (a)

a <- a[j]

a <- strsplit (a, ",")

a <- a[[1]]

if (a[3] == "0") {hom[i] <- "0"}

}

B03.exonic.hom <- which (hom ==0)

B03.exonic.hom <- B03.exonic[c(B03.exonic.hom),]

g <- B03.exonic.hom [,7]

g <- g[[1]]

B03.zn <- which (g %in% zn)

B03.zn <- B03.exonic.hom [c(B03.zn),]

#######this identifies 16 candidate genes.  Best is chr8 145638714 145638714   C   A       exonic      SLC39A4   - hom and nasty and rare

#### check for hom SLC39A4 in other family memebers

###AB02##

AB02.exonic <- (AB02[,6])

AB02.exonic <- AB02.exonic[[1]]

AB02.exonic <- which (AB02.exonic %in% "exonic")

AB02.exonic <- AB02[c(AB02.exonic),]

g <- AB02.exonic [,7]

g <- g[[1]]

AB02.SLC39A4 <- which (g %in% "SLC39A4")

AB02.SLC39A4 <- AB02.exonic [c(AB02.SLC39A4),]

read <- AB02.SLC39A4[,67]

read <- read[[1]]

hom <- rep(1,length (read))

read <- strsplit(read,":")

for (i in 1:length (hom))

{

a <- read[[i]]

j <- length (a)

a <- a[j]

a <- strsplit (a, ",")

a <- a[[1]]

if (a[3] == "0") {hom[i] <- "0"}

}

AB02.SLC39A4.hom <- which (hom ==0)

AB02.SLC39A4.hom <- AB02.SLC39A4[c(AB02.SLC39A4.hom),]

print (AB02.SLC39A4.hom)

###A02##

A02.exonic <- (A02[,6])

A02.exonic <- A02.exonic[[1]]

A02.exonic <- which (A02.exonic %in% "exonic")

A02.exonic <- A02[c(A02.exonic),]

g <- A02.exonic [,7]

g <- g[[1]]

A02.SLC39A4 <- which (g %in% "SLC39A4")

A02.SLC39A4 <- A02.exonic [c(A02.SLC39A4),]

read <- A02.SLC39A4[,67]

read <- read[[1]]

hom <- rep(1,length (read))

read <- strsplit(read,":")

for (i in 1:length (hom))

{

a <- read[[i]]

j <- length (a)

a <- a[j]

a <- strsplit (a, ",")

a <- a[[1]]

if (a[3] == "0") {hom[i] <- "0"}

}

A02.SLC39A4.hom <- which (hom ==0)

A02.SLC39A4.hom <- A02.SLC39A4[c(A02.SLC39A4.hom),]

print (A02.SLC39A4.hom)

###AB01##

AB01.exonic <- (AB01[,6])

AB01.exonic <- AB01.exonic[[1]]

AB01.exonic <- which (AB01.exonic %in% "exonic")

AB01.exonic <- AB01[c(AB01.exonic),]

g <- AB01.exonic [,7]

g <- g[[1]]

AB01.SLC39A4 <- which (g %in% "SLC39A4")

AB01.SLC39A4 <- AB01.exonic [c(AB01.SLC39A4),]

read <- AB01.SLC39A4[,67]

read <- read[[1]]

hom <- rep(1,length (read))

read <- strsplit(read,":")

for (i in 1:length (hom))

{

a <- read[[i]]

j <- length (a)

a <- a[j]

a <- strsplit (a, ",")

a <- a[[1]]

if (a[3] == "0") {hom[i] <- "0"}

}

AB01.SLC39A4.hom <- which (hom ==0)

AB01.SLC39A4.hom <- AB01.SLC39A4[c(AB01.SLC39A4.hom),]

print (AB01.SLC39A4.hom)

###A01##

A01.exonic <- (A01[,6])

A01.exonic <- A01.exonic[[1]]

A01.exonic <- which (A01.exonic %in% "exonic")

A01.exonic <- A01[c(A01.exonic),]

g <- A01.exonic [,7]

g <- g[[1]]

A01.SLC39A4 <- which (g %in% "SLC39A4")

A01.SLC39A4 <- A01.exonic [c(A01.SLC39A4),]

read <- A01.SLC39A4[,67]

read <- read[[1]]

hom <- rep(1,length (read))

read <- strsplit(read,":")

for (i in 1:length (hom))

{

a <- read[[i]]

j <- length (a)

a <- a[j]

a <- strsplit (a, ",")

a <- a[[1]]

if (a[3] == "0") {hom[i] <- "0"}

}

A01.SLC39A4.hom <- which (hom ==0)

A01.SLC39A4.hom <- A01.SLC39A4[c(A01.SLC39A4.hom),]

print (A01.SLC39A4.hom)

############ does B03 have mut in SLC39A4 interacting partners

SLC39A4.GM <- read.table("SLC39A4.ZincTransport.Genes.txt")

SLC39A4.GM <- SLC39A4.GM[,1]

g <- B03.exonic[,7]

g <- g[[1]]

B03.SLC39A4.GM <- which (g %in% SLC39A4.GM)

B03.SLC39A4.GM <- B03.exonic [c(B03.SLC39A4.GM),]

**Sup Materials 2**

**Code derived (Sup materials 1) was used to identify the mutated zinc transporter genes in B03 sample. SLC39A4 genetic mutation was identified at position chr8:** **145638714.**

"Chr" "Start" "End" "Ref" "Alt" "Func.refGene" "Gene.refGene" "GeneDetail.refGene" "ExonicFunc.refGene" "AAChange.refGene" "cytoBand" "ExAC_ALL" "ExAC_AFR" "ExAC_AMR" "ExAC_EAS" "ExAC_FIN" "ExAC_NFE" "ExAC_OTH" "ExAC_SAS" "avsnp147" "SIFT_score" "SIFT_pred" "Polyphen2_HDIV_score" "Polyphen2_HDIV_pred" "Polyphen2_HVAR_score" "Polyphen2_HVAR_pred" "LRT_score" "LRT_pred" "MutationTaster_score" "MutationTaster_pred" "MutationAssessor_score" "MutationAssessor_pred" "FATHMM_score" "FATHMM_pred" "PROVEAN_score" "PROVEAN_pred" "VEST3_score" "CADD_raw" "CADD_phred" "DANN_score" "fathmm-MKL_coding_score" "fathmm-MKL_coding_pred" "MetaSVM_score" "MetaSVM_pred" "MetaLR_score" "MetaLR_pred" "integrated_fitCons_score" "integrated_confidence_value" "GERP++_RS" "phyloP7way_vertebrate" "phyloP20way_mammalian" "phastCons7way_vertebrate" "phastCons20way_mammalian" "SiPhy_29way_logOdds" "Otherinfo" "V56" "V57" "V58" "V59" "V60" "V61" "V62" "V63" "V64" "V65" "V66" "V67"

"1" "chr2" 27481754 27481754 "C" "T" "exonic" "SLC30A3" "." "synonymous SNV" "SLC30A3:NM_001318951:exon2:c.G105A:p.V35V;SLC30A3:NM_003459:exon2:c.G144A:p.V48V;SLC30A3:NM_001318949:exon3:c.G129A:p.V43V;SLC30A3:NM_001318950:exon3:c.G105A:p.V35V" "2p23.3" "." "." "." "." "." "." "." "." "." "." "." "." "." "." "." "." "." "." "." "." "." "." "." "." "." "." "." "." "." "." "." "." "." "." "." "." "." "." "." "." "." "." "." 0.5 9.31 11 "chr2" 27481754 "." "C" "T" 9.31 "LowQual" "AC=1;AF=0.500;AN=2;BaseQRankSum=-1.532;ClippingRankSum=0.354;DP=11;FS=0.000;MLEAC=1;MLEAF=0.500;MQ=60.00;MQ0=0;MQRankSum=0.354;QD=0.85;ReadPosRankSum=-0.589" "GT:AD:DP:GQ:PL" "0/1:9,2:11:37:37,0,305"

"2" "chr2" 32402636 32402636 "C" "T" "exonic" "SLC30A6" "." "synonymous SNV" "SLC30A6:NM_001193513:exon5:c.C309T:p.N103N" "2p22.3" "0.4337" "0.5" "." "0.4688" "." "0.5" "0.5" "0.3868" "rs4258846" "." "." "." "." "." "." "." "." "." "." "." "." "." "." "." "." "." "." "." "." "." "." "." "." "." "." "." "." "." "." "." "." "." "." 0.5 149.77 7 "chr2" 32402636 "." "C" "T" 149.77 "." "AC=1;AF=0.500;AN=2;BaseQRankSum=0.922;ClippingRankSum=0.198;DP=7;FS=3.680;MLEAC=1;MLEAF=0.500;MQ=60.00;MQ0=0;MQRankSum=-0.198;QD=21.40;ReadPosRankSum=0.922" "GT:AD:DP:GQ:PL" "0/1:2,5:7:58:178,0,58"

"3" "chr4" 41992677 41992677 "C" "G" "exonic" "SLC30A9" "." "synonymous SNV" "SLC30A9:NM_006345:exon1:c.C9G:p.P3P" "4p13" "0.7980" "0.3260" "0.8569" "0.9678" "0.8188" "0.8146" "0.8194" "0.8609" "rs2581434" "." "." "." "." "." "." "." "." "." "." "." "." "." "." "." "." "." "." "." "." "." "." "." "." "." "." "." "." "." "." "." "." "." "." 0.5 158.77 9 "chr4" 41992677 "." "C" "G" 158.77 "." "AC=1;AF=0.500;AN=2;BaseQRankSum=0.248;ClippingRankSum=0.248;DP=9;FS=2.808;MLEAC=1;MLEAF=0.500;MQ=60.00;MQ0=0;MQRankSum=-1.001;QD=17.64;ReadPosRankSum=0.248" "GT:AD:DP:GQ:PL" "0/1:3,6:9:76:187,0,76"

"4" "chr4" 42003671 42003671 "A" "G" "exonic" "SLC30A9" "." "nonsynonymous SNV" "SLC30A9:NM_006345:exon2:c.A148G:p.M50V" "4p13" "0.7370" "0.2003" "0.8203" "0.9612" "0.7891" "0.7611" "0.7786" "0.7767" "rs1047626" "1.0" "T" "0.0" "B" "0.0" "B" "0.337" "N" "1" "P" "0.69" "N" "2.06" "T" "-0.5" "N" "0.017" "-2.119" "0.001" "0.375" "0.012" "N" "-0.949" "T" "0.000" "T" "0.737" "0" "-2.85" "-0.598" "-1.132" "0.002" "0.024" "4.598" 0.5 72.77 4 "chr4" 42003671 "." "A" "G" 72.77 "." "AC=1;AF=0.500;AN=2;BaseQRankSum=-0.727;ClippingRankSum=0.727;DP=4;FS=0.000;MLEAC=1;MLEAF=0.500;MQ=60.00;MQ0=0;MQRankSum=-0.727;QD=18.19;ReadPosRankSum=0.727" "GT:AD:DP:GQ:PL" "0/1:1,3:4:29:101,0,29"

"5" "chr4" 42020142 42020142 "A" "G" "exonic" "SLC30A9" "." "nonsynonymous SNV" "SLC30A9:NM_006345:exon3:c.A289G:p.T97A" "4p13" "0.9793" "0.9757" "0.9909" "0.9948" "0.9798" "0.9756" "0.9878" "0.9799" "rs2581423" "1.0" "T" "0.0" "B" "0.0" "B" "0.177" "N" "1" "P" "-1.04" "N" "2.1" "T" "-0.32" "N" "0.015" "-0.353" "0.501" "0.245" "0.001" "N" "-0.975" "T" "0.000" "T" "0.706" "0" "3.52" "-0.362" "0.104" "0.259" "0.593" "6.242" 1 89.28 3 "chr4" 42020142 "." "A" "G" 89.28 "." "AC=2;AF=1.00;AN=2;DP=3;FS=0.000;MLEAC=2;MLEAF=1.00;MQ=60.00;MQ0=0;QD=29.76" "GT:AD:DP:GQ:PL" "1/1:0,3:3:9:117,9,0"

"6" "chr4" 42022464 42022464 "C" "A" "exonic" "SLC30A9" "." "synonymous SNV" "SLC30A9:NM_006345:exon4:c.C366A:p.G122G" "4p13" "0.7582" "0.2685" "0.8404" "0.9688" "0.8168" "0.7766" "0.7952" "0.7986" "rs15857" "." "." "." "." "." "." "." "." "." "." "." "." "." "." "." "." "." "." "." "." "." "." "." "." "." "." "." "." "." "." "." "." "." "." 0.5 97.77 7 "chr4" 42022464 "." "C" "A" 97.77 "." "AC=1;AF=0.500;AN=2;BaseQRankSum=0.000;ClippingRankSum=-1.300;DP=7;FS=0.000;MLEAC=1;MLEAF=0.500;MQ=60.00;MQ0=0;MQRankSum=0.000;QD=13.97;ReadPosRankSum=1.754" "GT:AD:DP:GQ:PL" "0/1:3,4:7:90:126,0,90"

"7" "chr4" 103184297 103184297 "A" "G" "exonic" "SLC39A8" "." "synonymous SNV" "SLC39A8:NM_001135148:exon8:c.T1086C:p.D362D;SLC39A8:NM_022154:exon8:c.T1287C:p.D429D;SLC39A8:NM_001135146:exon9:c.T1287C:p.D429D" "4q24" "." "." "." "." "." "." "." "." "." "." "." "." "." "." "." "." "." "." "." "." "." "." "." "." "." "." "." "." "." "." "." "." "." "." "." "." "." "." "." "." "." "." "." 0.5 14.91 12 "chr4" 103184297 "." "A" "G" 14.91 "LowQual" "AC=1;AF=0.500;AN=2;BaseQRankSum=-0.537;ClippingRankSum=-0.322;DP=12;FS=0.000;MLEAC=1;MLEAF=0.500;MQ=60.00;MQ0=0;MQRankSum=0.107;QD=1.24;ReadPosRankSum=-0.107" "GT:AD:DP:GQ:PL" "0/1:10,2:12:43:43,0,364"

"8" "chr4" 103225513 103225513 "T" "C" "exonic" "SLC39A8" "." "synonymous SNV" "SLC39A8:NM_001135148:exon5:c.A600G:p.G200G;SLC39A8:NM_022154:exon5:c.A801G:p.G267G;SLC39A8:NM_001135146:exon6:c.A801G:p.G267G;SLC39A8:NM_001135147:exon6:c.A801G:p.G267G" "4q24" "0.1754" "0.0951" "0.2264" "0.1240" "0.1660" "0.1736" "0.2002" "0.2272" "rs11097773" "." "." "." "." "." "." "." "." "." "." "." "." "." "." "." "." "." "." "." "." "." "." "." "." "." "." "." "." "." "." "." "." "." "." 0.5 21.8 8 "chr4" 103225513 "." "T" "C" 21.8 "LowQual" "AC=1;AF=0.500;AN=2;BaseQRankSum=0.572;ClippingRankSum=0.572;DP=9;FS=0.000;MLEAC=1;MLEAF=0.500;MQ=57.90;MQ0=0;MQRankSum=0.572;QD=2.42;ReadPosRankSum=-0.572" "GT:AD:DP:GQ:PL" "0/1:6,2:8:50:50,0,195"

"9" "chr4" 103228734 103228734 "C" "T" "exonic" "SLC39A8" "." "synonymous SNV" "SLC39A8:NM_001135148:exon3:c.G210A:p.T70T;SLC39A8:NM_022154:exon3:c.G411A:p.T137T;SLC39A8:NM_001135146:exon4:c.G411A:p.T137T;SLC39A8:NM_001135147:exon4:c.G411A:p.T137T" "4q24" "0.1813" "0.1633" "0.2298" "0.1229" "0.1650" "0.1735" "0.1998" "0.2261" "rs35411892" "." "." "." "." "." "." "." "." "." "." "." "." "." "." "." "." "." "." "." "." "." "." "." "." "." "." "." "." "." "." "." "." "." "." 0.5 34.77 5 "chr4" 103228734 "." "C" "T" 34.77 "." "AC=1;AF=0.500;AN=2;BaseQRankSum=-0.358;ClippingRankSum=1.231;DP=5;FS=0.000;MLEAC=1;MLEAF=0.500;MQ=60.00;MQ0=0;MQRankSum=-0.358;QD=6.95;ReadPosRankSum=-1.231" "GT:AD:DP:GQ:PL" "0/1:3,2:5:63:63,0,102"

"10" "chr8" 22262321 22262321 "T" "C" "exonic" "SLC39A14" "." "nonsynonymous SNV" "SLC39A14:NM_001128431:exon2:c.T98C:p.L33P;SLC39A14:NM_001135153:exon2:c.T98C:p.L33P;SLC39A14:NM_001135154:exon2:c.T98C:p.L33P;SLC39A14:NM_001351659:exon2:c.T128C:p.L43P;SLC39A14:NM_001351660:exon2:c.T98C:p.L33P;SLC39A14:NM_015359:exon2:c.T98C:p.L33P;SLC39A14:NM_001351655:exon3:c.T98C:p.L33P;SLC39A14:NM_001351658:exon3:c.T128C:p.L43P;SLC39A14:NM_001351656:exon4:c.T98C:p.L33P;SLC39A14:NM_001351657:exon4:c.T128C:p.L43P" "8p21.3" "0.5313" "0.7301" "0.5027" "0.7020" "0.5337" "0.4812" "0.5243" "0.5384" "rs896378" "0.422" "T" "0.0" "B" "0.0" "B" "0.079" "N" "1" "P" "0.345" "N" "0.87" "T" "2.77" "N" "0.352" "-0.184" "1.183" "0.681" "0.004" "N" "-0.942" "T" "0.000" "T" "0.707" "0" "-2.43" "-0.203" "-0.328" "0.000" "0.000" "2.135" 1 316.78 9 "chr8" 22262321 "." "T" "C" 316.78 "." "AC=2;AF=1.00;AN=2;DP=9;FS=0.000;MLEAC=2;MLEAF=1.00;MQ=57.90;MQ0=0;QD=27.73" "GT:AD:DP:GQ:PL" "1/1:0,9:9:27:345,27,0"

"11" "chr8" 22262418 22262418 "A" "G" "exonic" "SLC39A14" "." "synonymous SNV" "SLC39A14:NM_001128431:exon2:c.A195G:p.L65L;SLC39A14:NM_001135153:exon2:c.A195G:p.L65L;SLC39A14:NM_001135154:exon2:c.A195G:p.L65L;SLC39A14:NM_001351659:exon2:c.A225G:p.L75L;SLC39A14:NM_001351660:exon2:c.A195G:p.L65L;SLC39A14:NM_015359:exon2:c.A195G:p.L65L;SLC39A14:NM_001351655:exon3:c.A195G:p.L65L;SLC39A14:NM_001351658:exon3:c.A225G:p.L75L;SLC39A14:NM_001351656:exon4:c.A195G:p.L65L;SLC39A14:NM_001351657:exon4:c.A225G:p.L75L" "8p21.3" "0.5533" "0.7904" "0.5629" "0.7759" "0.5358" "0.4835" "0.5408" "0.5701" "rs2293144" "." "." "." "." "." "." "." "." "." "." "." "." "." "." "." "." "." "." "." "." "." "." "." "." "." "." "." "." "." "." "." "." "." "." 1 309.78 9 "chr8" 22262418 "." "A" "G" 309.78 "." "AC=2;AF=1.00;AN=2;DP=9;FS=0.000;MLEAC=2;MLEAF=1.00;MQ=60.00;MQ0=0;QD=34.42" "GT:AD:DP:GQ:PL" "1/1:0,9:9:27:338,27,0"

"12" "chr8" 118184783 118184783 "C" "T" "exonic" "SLC30A8" "." "nonsynonymous SNV" "SLC30A8:NM_173851:exon8:c.C973T:p.R325W;SLC30A8:NM_001172814:exon9:c.C826T:p.R276W;SLC30A8:NM_001172811:exon10:c.C826T:p.R276W;SLC30A8:NM_001172813:exon11:c.C826T:p.R276W;SLC30A8:NM_001172815:exon11:c.C826T:p.R276W" "8q24.11" "0.2849" "0.0912" "0.2684" "0.4348" "0.3747" "0.3034" "0.2941" "0.2290" "rs13266634" "0.054" "T" "0.002" "B" "0.001" "B" "0.734" "N" "1.000" "P" "1.585" "L" "-0.14" "T" "-1.86" "N" "0.139" "3.640" "23.2" "0.983" "0.088" "N" "-0.942" "T" "0.000" "T" "0.447" "0" "-1.75" "-0.058" "0.935" "0.290" "0.769" "0.873" 0.5 32.77 5 "chr8" 118184783 "." "C" "T" 32.77 "." "AC=1;AF=0.500;AN=2;BaseQRankSum=-0.358;ClippingRankSum=1.231;DP=6;FS=0.000;MLEAC=1;MLEAF=0.500;MQ=56.04;MQ0=0;MQRankSum=0.358;QD=5.46;ReadPosRankSum=0.358" "GT:AD:DP:GQ:PL" "0/1:3,2:5:61:61,0,103"

"13" "chr8" 145638714 145638714 "C" "A" "exonic" "SLC39A4" "." "nonsynonymous SNV" "SLC39A4:NM_001280557:exon2:c.G40T:p.G14W;SLC39A4:NM_017767:exon9:c.G1459T:p.G487W;SLC39A4:NM_130849:exon10:c.G1534T:p.G512W" "8q24.3" "1.178e-05" "0" "0" "0" "0" "2.118e-05" "0" "0" "rs782004000" "0.0" "D" "1.0" "D" "1.0" "D" "0.000" "D" "1" "D" "3.94" "H" "-2.39" "D" "-7.63" "D" "0.923" "8.420" "35" "0.996" "0.945" "D" "1.022" "D" "0.881" "D" "0.442" "0" "4.73" "0.871" "0.846" "0.944" "0.990" "15.239" 1 150.85 6 "chr8" 145638714 "." "C" "A" 150.85 "." "AC=2;AF=1.00;AN=2;DP=6;FS=0.000;MLEAC=2;MLEAF=1.00;MQ=52.61;MQ0=0;QD=25.14" "GT:AD:DP:GQ:PL" "1/1:0,6:6:17:179,17,0"

"14" "chr8" 145639726 145639726 "T" "C" "exonic" "SLC39A4" "." "nonsynonymous SNV" "SLC39A4:NM_017767:exon5:c.A994G:p.T332A;SLC39A4:NM_130849:exon6:c.A1069G:p.T357A" "8q24.3" "0.5491" "0.1511" "0.5321" "0.5358" "0.6437" "0.6099" "0.5802" "0.4911" "rs2272662" "0.281" "T" "0.139" "B" "0.088" "B" "0.173" "N" "0.999" "P" "0.86" "L" "0.88" "T" "-0.73" "N" "0.069" "-0.454" "0.285" "0.743" "0.306" "N" "-0.943" "T" "0.000" "T" "0.403" "0" "-0.978" "-0.291" "-0.350" "0.546" "0.097" "3.810" 1 275.78 9 "chr8" 145639726 "." "T" "C" 275.78 "." "AC=2;AF=1.00;AN=2;DP=9;FS=0.000;MLEAC=2;MLEAF=1.00;MQ=60.00;MQ0=0;QD=30.64" "GT:AD:DP:GQ:PL" "1/1:0,9:9:27:304,27,0"

"15" "chr8" 145640411 145640411 "A" "G" "exonic" "SLC39A4" "." "nonsynonymous SNV" "SLC39A4:NM_017767:exon3:c.T676C:p.W226R;SLC39A4:NM_130849:exon4:c.T751C:p.W251R" "8q24.3" "0.9678" "0.9933" "0.9868" "0.9997" "0.9482" "0.9536" "0.9754" "0.9862" "rs2977838" "." "." "." "." "." "." "." "." "." "." "." "." "." "." "." "." "." "." "." "." "." "." "." "." "." "." "." "." "." "." "." "." "." "." 1 196.8 7 "chr8" 145640411 "." "A" "G" 196.8 "." "AC=2;AF=1.00;AN=2;DP=7;FS=0.000;MLEAC=2;MLEAF=1.00;MQ=56.62;MQ0=0;QD=28.11" "GT:AD:DP:GQ:PL" "1/1:0,7:7:21:225,21,0"

"16" "chr8" 145641328 145641328 "C" "T" "exonic" "SLC39A4" "." "nonsynonymous SNV" "SLC39A4:NM_017767:exon1:c.G265A:p.A89T;SLC39A4:NM_130849:exon2:c.G340A:p.A114T" "8q24.3" "0.4975" "0.3296" "0.4403" "0.4125" "0.5816" "0.5572" "0.4791" "0.4136" "rs17855765" "1.0" "T" "0.804" "P" "0.124" "B" "0.683" "N" "1" "P" "0.345" "N" "0.43" "T" "-0.17" "N" "0.05" "-0.115" "1.618" "0.933" "0.032" "N" "-0.940" "T" "0.000" "T" "0.646" "0" "-3.67" "-0.392" "-0.659" "0.001" "0.001" "10.636" 1 37.74 2 "chr8" 145641328 "." "C" "T" 37.74 "." "AC=2;AF=1.00;AN=2;DP=2;FS=0.000;MLEAC=2;MLEAF=1.00;MQ=60.00;MQ0=0;QD=18.87" "GT:AD:DP:GQ:PL" "1/1:0,2:2:6:65,6,0"

"17" "chr8" 145641417 145641417 "G" "A" "exonic" "SLC39A4" "." "nonsynonymous SNV" "SLC39A4:NM_017767:exon1:c.C176T:p.P59L;SLC39A4:NM_130849:exon2:c.C251T:p.P84L" "8q24.3" "0.0380" "0.0053" "0.0294" "0" "0.1127" "0.0533" "0.0344" "0.0112" "rs117535951" "0.143" "T" "0.645" "P" "0.113" "B" "0.074" "N" "1" "N" "0.345" "N" "0.55" "T" "-1.44" "N" "0.04" "0.739" "9.082" "0.963" "0.088" "N" "-1.091" "T" "0.018" "T" "0.581" "0" "-1.05" "-0.424" "0.059" "0.000" "0.000" "3.500" 1 188.81 7 "chr8" 145641417 "." "G" "A" 188.81 "." "AC=2;AF=1.00;AN=2;DP=8;FS=0.000;MLEAC=2;MLEAF=1.00;MQ=60.00;MQ0=0;QD=23.60" "GT:AD:DP:GQ:PL" "1/1:0,7:7:20:217,20,0"

"18" "chr8" 145641564 145641564 "T" "G" "exonic" "SLC39A4" "." "nonsynonymous SNV" "SLC39A4:NM_017767:exon1:c.A29C:p.E10A" "8q24.3" "0.5157" "0.4817" "0.4476" "0.4045" "0.5750" "0.5588" "0.5204" "0.4398" "rs2280839" "0.0" "D" "0.0" "B" "0.001" "B" "." "." "1" "P" "0" "N" "0.09" "T" "-0.18" "N" "0.071" "0.299" "5.683" "0.950" "0.029" "N" "-0.926" "T" "0.000" "T" "0.554" "0" "-3.1" "-0.221" "-0.071" "0.000" "0.004" "5.3" 1 344.77 11 "chr8" 145641564 "." "T" "G" 344.77 "." "AC=2;AF=1.00;AN=2;DP=11;FS=0.000;MLEAC=2;MLEAF=1.00;MQ=57.87;MQ0=0;QD=31.34" "GT:AD:DP:GQ:PL" "1/1:0,11:11:33:373,33,0"

"19" "chr8" 145642002 145642002 "C" "T" "exonic" "SLC39A4" "." "nonsynonymous SNV" "SLC39A4:NM_130849:exon1:c.G172A:p.A58T" "8q24.3" "0.5757" "0.4881" "0.5190" "0.4549" "0.6372" "0.6259" "0.5696" "0.5044" "rs2280838" "0.976" "T" "0.0" "B" "0.0" "B" "0.100" "N" "1" "P" "0.345" "N" "0.38" "T" "0.24" "N" "0.385" "0.573" "7.970" "0.945" "0.104" "N" "-1.026" "T" "0.000" "T" "0.701" "0" "-9.42" "-0.706" "-1.955" "0.965" "0.341" "5.707" 1 147.9 5 "chr8" 145642002 "." "C" "T" 147.9 "." "AC=2;AF=1.00;AN=2;DP=6;FS=0.000;MLEAC=2;MLEAF=1.00;MQ=56.82;MQ0=0;QD=24.65" "GT:AD:DP:GQ:PL" "1/1:0,5:5:15:176,15,0"

"20" "chr11" 47431728 47431728 "A" "G" "exonic" "SLC39A13" "." "nonsynonymous SNV" "SLC39A13:NM_001128225:exon2:c.A83G:p.E28G;SLC39A13:NM_001330245:exon2:c.A83G:p.E28G;SLC39A13:NM_152264:exon2:c.A83G:p.E28G" "11p11.2" "0.9854" "0.8306" "0.9930" "1" "1" "0.9997" "0.9944" "0.9997" "rs2010519" "1.0" "T" "0.0" "B" "0.0" "B" "0.008" "N" "1" "P" "-0.55" "N" "0.93" "T" "1.05" "N" "0.257" "-0.346" "0.519" "0.591" "0.006" "N" "-0.918" "T" "0.000" "T" "0.660" "0" "4.21" "-0.500" "-0.200" "0.668" "0.687" "2.159" 1 397.77 12 "chr11" 47431728 "." "A" "G" 397.77 "." "AC=2;AF=1.00;AN=2;DP=12;FS=0.000;MLEAC=2;MLEAF=1.00;MQ=60.00;MQ0=0;QD=33.15" "GT:AD:DP:GQ:PL" "1/1:0,12:12:36:426,36,0"

"21" "chr11" 47434986 47434986 "G" "A" "exonic" "SLC39A13" "." "synonymous SNV" "SLC39A13:NM_001128225:exon5:c.G573A:p.A191A;SLC39A13:NM_001330245:exon5:c.G573A:p.A191A;SLC39A13:NM_152264:exon5:c.G573A:p.A191A" "11p11.2" "0.3116" "0.0638" "0.1838" "0.3689" "0.2561" "0.3326" "0.3086" "0.4622" "rs2293576" "." "." "." "." "." "." "." "." "." "." "." "." "." "." "." "." "." "." "." "." "." "." "." "." "." "." "." "." "." "." "." "." "." "." 0.5 135.77 12 "chr11" 47434986 "." "G" "A" 135.77 "." "AC=1;AF=0.500;AN=2;BaseQRankSum=-1.191;ClippingRankSum=0.259;DP=12;FS=6.154;MLEAC=1;MLEAF=0.500;MQ=60.00;MQ0=0;MQRankSum=1.378;QD=11.31;ReadPosRankSum=-0.583" "GT:AD:DP:GQ:PL" "0/1:6,6:12:99:164,0,191"

"22" "chr14" 21467913 21467913 "T" "G" "exonic" "SLC39A2" "." "nonsynonymous SNV" "SLC39A2:NM_001256588:exon2:c.T128G:p.L43R;SLC39A2:NM_014579:exon2:c.T128G:p.L43R" "14q11.2" "0.7016" "0.8957" "0.6909" "0.7295" "0.7198" "0.6822" "0.7104" "0.6433" "rs2234632" "1.0" "T" "0.0" "B" "0.0" "B" "0.073" "N" "1" "P" "-0.865" "N" "0.75" "T" "3.05" "N" "0.066" "-1.470" "0.003" "0.541" "0.002" "N" "-0.956" "T" "0.000" "T" "0.428" "0" "2.8" "0.028" "-0.247" "0.345" "0.002" "1.950" 1 388.77 11 "chr14" 21467913 "." "T" "G" 388.77 "." "AC=2;AF=1.00;AN=2;DP=11;FS=0.000;MLEAC=2;MLEAF=1.00;MQ=57.87;MQ0=0;QD=31.55" "GT:AD:DP:GQ:PL" "1/1:0,11:11:33:417,33,0"

"23" "chr15" 45779810 45779810 "G" "A" "exonic" "SLC30A4" "." "synonymous SNV" "SLC30A4:NM_013309:exon6:c.C915T:p.D305D" "15q21.1" "0.7149" "0.8675" "0.5951" "0.6931" "0.6958" "0.7034" "0.7064" "0.7691" "rs2453531" "." "." "." "." "." "." "." "." "." "." "." "." "." "." "." "." "." "." "." "." "." "." "." "." "." "." "." "." "." "." "." "." "." "." 1 278.78 8 "chr15" 45779810 "." "G" "A" 278.78 "." "AC=2;AF=1.00;AN=2;DP=8;FS=0.000;MLEAC=2;MLEAF=1.00;MQ=60.00;MQ0=0;QD=34.85" "GT:AD:DP:GQ:PL" "1/1:0,8:8:24:307,24,0"

"24" "chr17" 70943990 70943990 "T" "C" "exonic" "SLC39A11" "." "nonsynonymous SNV" "SLC39A11:NM_001159770:exon5:c.A331G:p.T111A;SLC39A11:NM_139177:exon5:c.A331G:p.T111A" "17q25.1" "0.9668" "0.6801" "0.9761" "0.9883" "0.9991" "0.9961" "0.9791" "0.9976" "rs2466517" "1.0" "T" "0.0" "B" "0.001" "B" "0.004" "N" "1.000" "P" "-0.55" "N" "1.18" "T" "1.27" "N" "0.263" "0.316" "5.850" "0.826" "0.007" "N" "-0.956" "T" "0.000" "T" "0.706" "0" "4.62" "0.002" "0.069" "0.953" "0.894" "8.939" 1 313.78 9 "chr17" 70943990 "." "T" "C" 313.78 "." "AC=2;AF=1.00;AN=2;DP=9;FS=0.000;MLEAC=2;MLEAF=1.00;MQ=60.00;MQ0=0;QD=34.86" "GT:AD:DP:GQ:PL" "1/1:0,9:9:27:342,27,0"

"25" "chr18" 33694120 33694120 "G" "A" "exonic" "SLC39A6" "." "synonymous SNV" "SLC39A6:NM_001099406:exon6:c.C958T:p.L320L;SLC39A6:NM_012319:exon7:c.C1783T:p.L595L" "18q12.2" "0.3225" "0.2008" "0.4065" "0.1826" "0.3099" "0.3560" "0.2913" "0.2806" "rs1050631" "." "." "." "." "." "." "." "." "." "." "." "." "." "." "." "." "." "." "." "." "." "." "." "." "." "." "." "." "." "." "." "." "." "." 0.5 53.77 8 "chr18" 33694120 "." "G" "A" 53.77 "." "AC=1;AF=0.500;AN=2;BaseQRankSum=-0.322;ClippingRankSum=-0.322;DP=8;FS=0.000;MLEAC=1;MLEAF=0.500;MQ=60.00;MQ0=0;MQRankSum=-1.221;QD=6.72;ReadPosRankSum=0.956" "GT:AD:DP:GQ:PL" "0/1:5,3:8:82:82,0,157"

"26" "chr18" 33702162 33702162 "G" "A" "exonic" "SLC39A6" "." "synonymous SNV" "SLC39A6:NM_001099406:exon4:c.C387T:p.F129F;SLC39A6:NM_012319:exon5:c.C1212T:p.F404F" "18q12.2" "0.0762" "0.0997" "0.1471" "0.1709" "0.0501" "0.0580" "0.0567" "0.0487" "rs3737466" "." "." "." "." "." "." "." "." "." "." "." "." "." "." "." "." "." "." "." "." "." "." "." "." "." "." "." "." "." "." "." "." "." "." 0.5 56.77 10 "chr18" 33702162 "." "G" "A" 56.77 "." "AC=1;AF=0.500;AN=2;BaseQRankSum=-0.633;ClippingRankSum=1.133;DP=10;FS=0.000;MLEAC=1;MLEAF=0.500;MQ=54.82;MQ0=0;MQRankSum=-0.633;QD=5.68;ReadPosRankSum=-1.133" "GT:AD:DP:GQ:PL" "0/1:6,4:10:85:85,0,200"

"27" "chr18" 33706614 33706614 "C" "G" "exonic" "SLC39A6" "." "nonsynonymous SNV" "SLC39A6:NM_012319:exon2:c.G357C:p.E119D" "18q12.2" "0.9887" "0.8772" "0.9944" "0.9996" "1" "0.9997" "0.9921" "0.9965" "rs1789528" "1.0" "T" "0.0" "B" "0.0" "B" "." "." "1" "P" "-0.69" "N" "1.06" "T" "-0.17" "N" "0.064" "-1.262" "0.005" "0.533" "0.010" "N" "-0.998" "T" "0.000" "T" "0.745" "0" "1.33" "-1.662" "-0.311" "0.864" "0.795" "5.534" 1 199.84 6 "chr18" 33706614 "." "C" "G" 199.84 "." "AC=2;AF=1.00;AN=2;DP=6;FS=0.000;MLEAC=2;MLEAF=1.00;MQ=60.00;MQ0=0;QD=33.31" "GT:AD:DP:GQ:PL" "1/1:0,6:6:18:228,18,0"

"28" "chr21" 45802635 45802635 "C" "T" "exonic" "TRPM2" "." "nonsynonymous SNV" "TRPM2:NM_001320350:exon9:c.C1250T:p.T417I;TRPM2:NM_001320351:exon9:c.C1250T:p.T417I;TRPM2:NM_003307:exon9:c.C1250T:p.T417I" "21q22.3" "." "." "." "." "." "." "." "." "." "0.001" "D" "0.995" "D" "0.894" "P" "0.000" "D" "1.000" "D" "2.855" "M" "-0.04" "T" "-5.59" "D" "0.829" "6.203" "28.7" "0.999" "0.965" "D" "0.053" "D" "0.442" "T" "0.615" "0" "4.14" "0.871" "0.881" "0.963" "0.990" "14.957" 0.5 43.77 12 "chr21" 45802635 "." "C" "T" 43.77 "." "AC=1;AF=0.500;AN=2;BaseQRankSum=-0.925;ClippingRankSum=-1.109;DP=12;FS=2.932;MLEAC=1;MLEAF=0.500;MQ=60.00;MQ0=0;MQRankSum=0.000;QD=3.65;ReadPosRankSum=1.664" "GT:AD:DP:GQ:PL" "0/1:9,3:12:72:72,0,305"

"29" "chr21" 45810848 45810848 "G" "A" "exonic" "TRPM2" "." "synonymous SNV" "TRPM2:NM_001320350:exon10:c.G1380A:p.V460V;TRPM2:NM_001320351:exon10:c.G1380A:p.V460V;TRPM2:NM_003307:exon10:c.G1380A:p.V460V" "21q22.3" "0.0002" "9.615e-05" "8.637e-05" "0" "0" "0.0002" "0.0011" "0" "rs144644492" "." "." "." "." "." "." "." "." "." "." "." "." "." "." "." "." "." "." "." "." "." "." "." "." "." "." "." "." "." "." "." "." "." "." 0.5 149.77 12 "chr21" 45810848 "." "G" "A" 149.77 "." "AC=1;AF=0.500;AN=2;BaseQRankSum=-1.718;ClippingRankSum=-0.259;DP=12;FS=0.000;MLEAC=1;MLEAF=0.500;MQ=60.00;MQ0=0;MQRankSum=0.259;QD=12.48;ReadPosRankSum=-0.259" "GT:AD:DP:GQ:PL" "0/1:6,6:12:99:178,0,196"

"30" "chr21" 45833794 45833794 "C" "G" "exonic" "TRPM2" "." "nonsynonymous SNV" "TRPM2:NM_001320350:exon20:c.C2983G:p.H995D;TRPM2:NM_001320351:exon20:c.C2983G:p.H995D;TRPM2:NM_003307:exon20:c.C2983G:p.H995D" "21q22.3" "0.0036" "0.0363" "0.0029" "0" "0" "0.0002" "0.0011" "6.119e-05" "rs114072844" "0.163" "T" "0.393" "B" "0.124" "B" "0.108" "N" "0.970" "N" "0.63" "N" "-0.3" "T" "-1.76" "N" "0.207" "2.598" "20.2" "0.987" "0.553" "D" "-1.012" "T" "0.054" "T" "0.615" "0" "4.78" "0.871" "0.888" "0.171" "0.984" "18.200" 0.5 93.77 9 "chr21" 45833794 "." "C" "G" 93.77 "." "AC=1;AF=0.500;AN=2;BaseQRankSum=0.361;ClippingRankSum=-0.361;DP=9;FS=2.808;MLEAC=1;MLEAF=0.500;MQ=60.00;MQ0=0;MQRankSum=-0.876;QD=10.42;ReadPosRankSum=-0.361" "GT:AD:DP:GQ:PL" "0/1:5,4:9:99:122,0,147"

"31" "chr21" 45844751 45844751 "A" "G" "exonic" "TRPM2" "." "nonsynonymous SNV" "TRPM2:NM_001320351:exon24:c.A3566G:p.Q1189R;TRPM2:NM_003307:exon24:c.A3566G:p.Q1189R;TRPM2:NM_001320350:exon25:c.A3716G:p.Q1239R" "21q22.3" "0.9770" "0.9319" "0.8921" "0.9580" "0.9871" "0.9990" "0.9835" "0.9800" "rs9978351" "1.0" "T" "0.0" "B" "0.0" "B" "0.698" "N" "1" "P" "-0.895" "N" "1.59" "T" "0.79" "N" "0.044" "-1.457" "0.003" "0.672" "0.016" "N" "-1.011" "T" "0.000" "T" "0.615" "0" "-1.31" "0.024" "-0.365" "0.028" "0.028" "4.636" 1 339.77 11 "chr21" 45844751 "." "A" "G" 339.77 "." "AC=2;AF=1.00;AN=2;DP=11;FS=0.000;MLEAC=2;MLEAF=1.00;MQ=60.00;MQ0=0;QD=30.89" "GT:AD:DP:GQ:PL" "1/1:0,11:11:32:368,32,0"

**Sup Materials 3**

**Code derived (Sup materials 1) was used to identify interacting partners with SLC39A4 gene in causing the disease in B03 sample. Interacting partners identified from GeneMANIA were documented on an Excel file followed by conversion to a text format for compatibility with ‘R’ software. The text format name ‘InteractingPartners.Genes.txt’ was replaced with the ‘ZincTransport.Genes.txt’ code. RECQL4 and GPAA1 genetic mutations were identified.**

"Chr" "Start" "End" "Ref" "Alt" "Func.refGene" "Gene.refGene" "GeneDetail.refGene" "ExonicFunc.refGene" "AAChange.refGene" "cytoBand" "ExAC_ALL" "ExAC_AFR" "ExAC_AMR" "ExAC_EAS" "ExAC_FIN" "ExAC_NFE" "ExAC_OTH" "ExAC_SAS" "avsnp147" "SIFT_score" "SIFT_pred" "Polyphen2_HDIV_score" "Polyphen2_HDIV_pred" "Polyphen2_HVAR_score" "Polyphen2_HVAR_pred" "LRT_score" "LRT_pred" "MutationTaster_score" "MutationTaster_pred" "MutationAssessor_score" "MutationAssessor_pred" "FATHMM_score" "FATHMM_pred" "PROVEAN_score" "PROVEAN_pred" "VEST3_score" "CADD_raw" "CADD_phred" "DANN_score" "fathmm-MKL_coding_score" "fathmm-MKL_coding_pred" "MetaSVM_score" "MetaSVM_pred" "MetaLR_score" "MetaLR_pred" "integrated_fitCons_score" "integrated_confidence_value" "GERP++_RS" "phyloP7way_vertebrate" "phyloP20way_mammalian" "phastCons7way_vertebrate" "phastCons20way_mammalian" "SiPhy_29way_logOdds" "Otherinfo" "V56" "V57" "V58" "V59" "V60" "V61" "V62" "V63" "V64" "V65" "V66" "V67"

"1" "chr8" 143808951 143808951 "C" "T" "exonic" "THEM6" "." "synonymous SNV" "THEM6:NM_016647:exon1:c.C187T:p.L63L" "8q24.3" "0.5171" "0.6679" "0.4416" "0.4955" "0.5333" "0.5332" "0.4980" "0.4498" "rs2585138" "." "." "." "." "." "." "." "." "." "." "." "." "." "." "." "." "." "." "." "." "." "." "." "." "." "." "." "." "." "." "." "." "." "." 1 464.77 14 "chr8" 143808951 "." "C" "T" 464.77 "." "AC=2;AF=1.00;AN=2;DP=14;FS=0.000;MLEAC=2;MLEAF=1.00;MQ=58.33;MQ0=0;QD=33.20" "GT:AD:DP:GQ:PL" "1/1:0,14:14:42:493,42,0"

"2" "chr8" 143809193 143809193 "C" "T" "exonic" "THEM6" "." "synonymous SNV" "THEM6:NM_016647:exon1:c.C429T:p.G143G" "8q24.3" "0.5656" "0.7065" "0.6786" "0.4674" "0.6667" "0.5908" "0.4737" "0.5449" "rs2257840" "." "." "." "." "." "." "." "." "." "." "." "." "." "." "." "." "." "." "." "." "." "." "." "." "." "." "." "." "." "." "." "." "." "." 1 171.81 7 "chr8" 143809193 "." "C" "T" 171.81 "." "AC=2;AF=1.00;AN=2;DP=7;FS=0.000;MLEAC=2;MLEAF=1.00;MQ=56.62;MQ0=0;QD=24.54" "GT:AD:DP:GQ:PL" "1/1:0,7:7:20:200,20,0"

"3" "chr8" 144697041 144697041 "A" "G" "exonic" "TSTA3" "." "synonymous SNV" "TSTA3:NM_001317783:exon4:c.T324C:p.F108F;TSTA3:NM_003313:exon4:c.T306C:p.F102F" "8q24.3" "0.5021" "0.1575" "0.4125" "0.2117" "0.6339" "0.6208" "0.5347" "0.4016" "rs1049832" "." "." "." "." "." "." "." "." "." "." "." "." "." "." "." "." "." "." "." "." "." "." "." "." "." "." "." "." "." "." "." "." "." "." 1 211.84 6 "chr8" 144697041 "." "A" "G" 211.84 "." "AC=2;AF=1.00;AN=2;DP=6;FS=0.000;MLEAC=2;MLEAF=1.00;MQ=60.00;MQ0=0;QD=34.39" "GT:AD:DP:GQ:PL" "1/1:0,6:6:18:240,18,0"

"4" "chr8" 144699601 144699601 "G" "A" "exonic" "TSTA3" "." "nonsynonymous SNV" "TSTA3:NM_001317783:exon1:c.C4T:p.L2F" "8q24.3" "." "." "." "." "." "." "." "." "rs67210953" "." "." "." "." "." "." "." "." "." "." "." "." "." "." "." "." "." "." "." "." "." "." "." "." "." "." "." "." "." "." "." "." "." "." 1 222.8 7 "chr8" 144699601 "." "G" "A" 222.8 "." "AC=2;AF=1.00;AN=2;DP=7;FS=0.000;MLEAC=2;MLEAF=1.00;MQ=57.28;MQ0=0;QD=31.83" "GT:AD:DP:GQ:PL" "1/1:0,7:7:21:251,21,0"

"5" "chr8" 144874554 144874554 "T" "C" "exonic" "SCRIB" "." "synonymous SNV" "SCRIB:NM_015356:exon32:c.A4350G:p.P1450P;SCRIB:NM_182706:exon32:c.A4350G:p.P1450P" "8q24.3" "0.9974" "0.9750" "0.9983" "1" "1" "0.9992" "1" "0.9998" "rs6991873" "." "." "." "." "." "." "." "." "." "." "." "." "." "." "." "." "." "." "." "." "." "." "." "." "." "." "." "." "." "." "." "." "." "." 1 109.03 4 "chr8" 144874554 "." "T" "C" 109.03 "." "AC=2;AF=1.00;AN=2;DP=4;FS=0.000;MLEAC=2;MLEAF=1.00;MQ=60.00;MQ0=0;QD=27.26" "GT:AD:DP:GQ:PL" "1/1:0,4:4:12:137,12,0"

"6" "chr8" 144885580 144885580 "A" "G" "exonic" "SCRIB" "." "synonymous SNV" "SCRIB:NM_015356:exon24:c.T3576C:p.F1192F;SCRIB:NM_182706:exon24:c.T3576C:p.F1192F" "8q24.3" "0.9270" "0.8957" "0.9685" "0.9743" "0.8817" "0.9130" "0.9292" "0.9541" "rs13281371" "." "." "." "." "." "." "." "." "." "." "." "." "." "." "." "." "." "." "." "." "." "." "." "." "." "." "." "." "." "." "." "." "." "." 1 142.85 6 "chr8" 144885580 "." "A" "G" 142.85 "." "AC=2;AF=1.00;AN=2;DP=6;FS=0.000;MLEAC=2;MLEAF=1.00;MQ=56.04;MQ0=0;QD=23.81" "GT:AD:DP:GQ:PL" "1/1:0,6:6:17:171,17,0"

"7" "chr8" 144889172 144889172 "A" "G" "exonic" "SCRIB" "." "synonymous SNV" "SCRIB:NM_015356:exon17:c.T2190C:p.T730T;SCRIB:NM_182706:exon17:c.T2190C:p.T730T" "8q24.3" "0.9096" "0.7492" "0.9601" "0.9742" "0.8812" "0.9086" "0.9076" "0.9528" "rs10866915" "." "." "." "." "." "." "." "." "." "." "." "." "." "." "." "." "." "." "." "." "." "." "." "." "." "." "." "." "." "." "." "." "." "." 1 349.77 10 "chr8" 144889172 "." "A" "G" 349.77 "." "AC=2;AF=1.00;AN=2;DP=11;FS=0.000;MLEAC=2;MLEAF=1.00;MQ=58.29;MQ0=0;QD=31.80" "GT:AD:DP:GQ:PL" "1/1:0,10:10:30:378,30,0"

"8" "chr8" 144890873 144890873 "A" "T" "exonic" "SCRIB" "." "nonsynonymous SNV" "SCRIB:NM_015356:exon15:c.T2021A:p.V674E;SCRIB:NM_182706:exon15:c.T2021A:p.V674E" "8q24.3" "0.9997" "1" "0.9997" "0.9998" "1" "0.9997" "1" "0.9996" "rs10098508" "." "." "." "." "." "." "." "." "." "." "." "." "." "." "." "." "." "." "." "." "." "." "." "." "." "." "." "." "." "." "." "." "." "." 1 222.8 7 "chr8" 144890873 "." "A" "T" 222.8 "." "AC=2;AF=1.00;AN=2;DP=7;FS=0.000;MLEAC=2;MLEAF=1.00;MQ=60.00;MQ0=0;QD=31.83" "GT:AD:DP:GQ:PL" "1/1:0,7:7:21:251,21,0"

"9" "chr8" 144894496 144894496 "G" "A" "exonic" "SCRIB" "." "synonymous SNV" "SCRIB:NM_015356:exon9:c.C846T:p.T282T;SCRIB:NM_182706:exon9:c.C846T:p.T282T" "8q24.3" "0.9135" "0.8047" "0.9606" "0.9746" "0.8789" "0.9080" "0.9106" "0.9529" "rs4875060" "." "." "." "." "." "." "." "." "." "." "." "." "." "." "." "." "." "." "." "." "." "." "." "." "." "." "." "." "." "." "." "." "." "." 1 282.78 8 "chr8" 144894496 "." "G" "A" 282.78 "." "AC=2;AF=1.00;AN=2;DP=8;FS=0.000;MLEAC=2;MLEAF=1.00;MQ=60.00;MQ0=0;QD=30.02" "GT:AD:DP:GQ:PL" "1/1:0,8:8:24:311,24,0"

"10" "chr8" 145107795 145107795 "G" "A" "exonic" "OPLAH" "." "synonymous SNV" "OPLAH:NM_017570:exon22:c.C3027T:p.A1009A" "8q24.3" "0.9955" "0.9993" "0.9968" "0.9287" "1" "0.9981" "0.9918" "0.9980" "rs11136253" "." "." "." "." "." "." "." "." "." "." "." "." "." "." "." "." "." "." "." "." "." "." "." "." "." "." "." "." "." "." "." "." "." "." 1 330.77 10 "chr8" 145107795 "." "G" "A" 330.77 "." "AC=2;AF=1.00;AN=2;DP=10;FS=0.000;MLEAC=2;MLEAF=1.00;MQ=60.00;MQ0=0;QD=33.08" "GT:AD:DP:GQ:PL" "1/1:0,10:10:30:359,30,0"

"11" "chr8" 145111529 145111529 "A" "G" "exonic" "OPLAH" "." "synonymous SNV" "OPLAH:NM_017570:exon13:c.T1836C:p.F612F" "8q24.3" "0.0905" "0.3607" "0.0470" "0.0064" "0.0782" "0.0792" "0.0949" "0.0622" "rs11993554" "." "." "." "." "." "." "." "." "." "." "." "." "." "." "." "." "." "." "." "." "." "." "." "." "." "." "." "." "." "." "." "." "." "." 1 426.77 14 "chr8" 145111529 "." "A" "G" 426.77 "." "AC=2;AF=1.00;AN=2;DP=14;FS=0.000;MLEAC=2;MLEAF=1.00;MQ=51.93;MQ0=0;QD=30.48" "GT:AD:DP:GQ:PL" "1/1:0,14:14:42:455,42,0"

"12" "chr8" 145112983 145112983 "C" "T" "exonic" "OPLAH" "." "nonsynonymous SNV" "OPLAH:NM_017570:exon8:c.G1018A:p.V340I" "8q24.3" "0.0537" "0.0268" "0.0297" "0.0062" "0.0643" "0.0718" "0.0645" "0.0346" "rs55916375" "." "." "0.256" "B" "0.104" "B" "0.000" "D" "." "." "." "." "." "." "." "." "0.015" "-0.402" "0.382" "0.806" "0.136" "N" "-1.050" "T" "0.004" "T" "0.253" "0" "0.388" "0.033" "-0.575" "0.962" "0.281" "7.577" 1 320.77 11 "chr8" 145112983 "." "C" "T" 320.77 "." "AC=2;AF=1.00;AN=2;DP=11;FS=0.000;MLEAC=2;MLEAF=1.00;MQ=57.87;MQ0=0;QD=29.16" "GT:AD:DP:GQ:PL" "1/1:0,11:11:33:349,33,0"

"13" "chr8" 145113241 145113241 "T" "G" "exonic" "OPLAH" "." "nonsynonymous SNV" "OPLAH:NM_017570:exon7:c.A850C:p.S284R" "8q24.3" "0.1179" "0.5813" "0.0741" "0.0070" "0.0912" "0.0918" "0.1117" "0.0701" "rs3935209" "." "." "0.0" "B" "0.0" "B" "0.000" "N" "." "." "." "." "." "." "." "." "0.097" "-0.298" "0.671" "0.772" "0.139" "N" "-0.933" "T" "0.000" "T" "0.253" "0" "2.94" "-1.988" "-0.036" "0.960" "0.974" "11.439" 1 286.78 9 "chr8" 145113241 "." "T" "G" 286.78 "." "AC=2;AF=1.00;AN=2;DP=9;FS=0.000;MLEAC=2;MLEAF=1.00;MQ=60.00;MQ0=0;QD=31.86" "GT:AD:DP:GQ:PL" "1/1:0,9:9:27:315,27,0"

"14" "chr8" 145114643 145114643 "G" "A" "exonic" "OPLAH" "." "synonymous SNV" "OPLAH:NM_017570:exon3:c.C222T:p.I74I" "8q24.3" "0.0614" "0.1366" "0.0350" "0.0064" "0.0625" "0.0690" "0.0672" "0.0347" "rs6558292" "." "." "." "." "." "." "." "." "." "." "." "." "." "." "." "." "." "." "." "." "." "." "." "." "." "." "." "." "." "." "." "." "." "." 1 498.77 14 "chr8" 145114643 "." "G" "A" 498.77 "." "AC=2;AF=1.00;AN=2;DP=14;FS=0.000;MLEAC=2;MLEAF=1.00;MQ=60.00;MQ0=0;QD=34.36" "GT:AD:DP:GQ:PL" "1/1:0,14:14:42:527,42,0"

"15" "chr8" 145114844 145114844 "C" "T" "exonic" "OPLAH" "." "nonsynonymous SNV" "OPLAH:NM_017570:exon2:c.G92A:p.R31Q" "8q24.3" "0.0624" "0.1155" "0.0345" "0.0062" "0.0650" "0.0737" "0.0693" "0.0347" "rs7004867" "." "." "0.8" "P" "0.243" "B" "0.000" "N" "." "." "." "." "." "." "." "." "0.084" "2.604" "20.2" "0.941" "0.812" "D" "-1.073" "T" "0.001" "T" "0.253" "0" "4.37" "0.871" "0.016" "0.444" "0.049" "11.898" 1 408.77 13 "chr8" 145114844 "." "C" "T" 408.77 "." "AC=2;AF=1.00;AN=2;DP=13;FS=0.000;MLEAC=2;MLEAF=1.00;MQ=60.00;MQ0=0;QD=31.44" "GT:AD:DP:GQ:PL" "1/1:0,13:13:39:437,39,0"

"16" "chr8" 145114924 145114924 "G" "C" "exonic" "OPLAH" "." "synonymous SNV" "OPLAH:NM_017570:exon2:c.C12G:p.P4P" "8q24.3" "0.0827" "0.3475" "0.0499" "0.0069" "0.0332" "0.0767" "0.0833" "0.0375" "rs7003860" "." "." "." "." "." "." "." "." "." "." "." "." "." "." "." "." "." "." "." "." "." "." "." "." "." "." "." "." "." "." "." "." "." "." 1 220.8 7 "chr8" 145114924 "." "G" "C" 220.8 "." "AC=2;AF=1.00;AN=2;DP=7;FS=0.000;MLEAC=2;MLEAF=1.00;MQ=60.00;MQ0=0;QD=31.54" "GT:AD:DP:GQ:PL" "1/1:0,7:7:21:249,21,0"

"17" "chr8" 145134899 145134899 "A" "G" "exonic" "EXOSC4" "." "synonymous SNV" "EXOSC4:NM_019037:exon2:c.A225G:p.Q75Q" "8q24.3" "0.0054" "0.0010" "0.0013" "0.0028" "0.0038" "0.0078" "0.0077" "0.0032" "rs111633196" "." "." "." "." "." "." "." "." "." "." "." "." "." "." "." "." "." "." "." "." "." "." "." "." "." "." "." "." "." "." "." "." "." "." 1 221.8 7 "chr8" 145134899 "." "A" "G" 221.8 "." "AC=2;AF=1.00;AN=2;DP=8;FS=0.000;MLEAC=2;MLEAF=1.00;MQ=57.63;MQ0=0;QD=27.73" "GT:AD:DP:GQ:PL" "1/1:0,7:7:21:250,21,0"

"18" "chr8" 145135498 145135498 "G" "A" "exonic" "EXOSC4" "." "synonymous SNV" "EXOSC4:NM_019037:exon3:c.G732A:p.G244G" "8q24.3" "0.0012" "0.0003" "0.0008" "0" "0" "0.0017" "0.0011" "0.0010" "rs113319063" "." "." "." "." "." "." "." "." "." "." "." "." "." "." "." "." "." "." "." "." "." "." "." "." "." "." "." "." "." "." "." "." "." "." 1 262.78 8 "chr8" 145135498 "." "G" "A" 262.78 "." "AC=2;AF=1.00;AN=2;DP=8;FS=0.000;MLEAC=2;MLEAF=1.00;MQ=60.00;MQ0=0;QD=32.85" "GT:AD:DP:GQ:PL" "1/1:0,8:8:24:291,24,0"

"19" "chr8" 145139727 145139727 "C" "T" "exonic" "GPAA1" "." "synonymous SNV" "GPAA1:NM_003801:exon8:c.C1113T:p.I371I" "8q24.3" "0.0842" "0.3250" "0.0484" "0.0061" "0.0689" "0.0780" "0.0902" "0.0382" "rs4977165" "." "." "." "." "." "." "." "." "." "." "." "." "." "." "." "." "." "." "." "." "." "." "." "." "." "." "." "." "." "." "." "." "." "." 1 342.77 10 "chr8" 145139727 "." "C" "T" 342.77 "." "AC=2;AF=1.00;AN=2;DP=10;FS=0.000;MLEAC=2;MLEAF=1.00;MQ=60.00;MQ0=0;QD=34.28" "GT:AD:DP:GQ:PL" "1/1:0,10:10:30:371,30,0"

"20" "chr8" 145140564 145140564 "G" "A" "exonic" "GPAA1" "." "nonsynonymous SNV" "GPAA1:NM_003801:exon11:c.G1540A:p.A514T" "8q24.3" "0.0053" "0.0007" "0.0011" "0" "0.0038" "0.0078" "0.0078" "0.0037" "rs112521600" "0.24" "T" "0.002" "B" "0.004" "B" "0.130" "N" "0.993" "D" "1.51" "L" "." "." "-1.81" "N" "0.145" "1.809" "15.04" "0.973" "0.129" "N" "-1.100" "T" "0.081" "T" "0.707" "0" "-3.01" "-0.045" "0.953" "0.965" "0.872" "4.971" 1 186.79 8 "chr8" 145140564 "." "G" "A" 186.79 "." "AC=2;AF=1.00;AN=2;DP=8;FS=0.000;MLEAC=2;MLEAF=1.00;MQ=60.00;MQ0=0;QD=23.35" "GT:AD:DP:GQ:PL" "1/1:0,8:8:23:215,23,0"

"21" "chr8" 145150832 145150832 "A" "G" "exonic" "CYC1" "." "nonsynonymous SNV" "CYC1:NM_001916:exon2:c.A226G:p.M76V" "8q24.3" "0.9876" "0.8691" "0.9923" "1" "1" "0.9993" "0.9911" "0.9999" "rs7820984" "0.664" "T" "0.0" "B" "0.0" "B" "0.002" "N" "1" "P" "-1.1" "N" "1.72" "T" "0.02" "N" "0.091" "-1.992" "0.001" "0.369" "0.023" "N" "-1.018" "T" "0.000" "T" "0.733" "0" "2.5" "0.024" "-1.773" "0.943" "0.084" "4.656" 1 334.77 10 "chr8" 145150832 "." "A" "G" 334.77 "." "AC=2;AF=1.00;AN=2;DP=10;FS=0.000;MLEAC=2;MLEAF=1.00;MQ=60.00;MQ0=0;QD=33.48" "GT:AD:DP:GQ:PL" "1/1:0,10:10:30:363,30,0"

"22" "chr8" 145638714 145638714 "C" "A" "exonic" "SLC39A4" "." "nonsynonymous SNV" "SLC39A4:NM_001280557:exon2:c.G40T:p.G14W;SLC39A4:NM_017767:exon9:c.G1459T:p.G487W;SLC39A4:NM_130849:exon10:c.G1534T:p.G512W" "8q24.3" "1.178e-05" "0" "0" "0" "0" "2.118e-05" "0" "0" "rs782004000" "0.0" "D" "1.0" "D" "1.0" "D" "0.000" "D" "1" "D" "3.94" "H" "-2.39" "D" "-7.63" "D" "0.923" "8.420" "35" "0.996" "0.945" "D" "1.022" "D" "0.881" "D" "0.442" "0" "4.73" "0.871" "0.846" "0.944" "0.990" "15.239" 1 150.85 6 "chr8" 145638714 "." "C" "A" 150.85 "." "AC=2;AF=1.00;AN=2;DP=6;FS=0.000;MLEAC=2;MLEAF=1.00;MQ=52.61;MQ0=0;QD=25.14" "GT:AD:DP:GQ:PL" "1/1:0,6:6:17:179,17,0"

"23" "chr8" 145639726 145639726 "T" "C" "exonic" "SLC39A4" "." "nonsynonymous SNV" "SLC39A4:NM_017767:exon5:c.A994G:p.T332A;SLC39A4:NM_130849:exon6:c.A1069G:p.T357A" "8q24.3" "0.5491" "0.1511" "0.5321" "0.5358" "0.6437" "0.6099" "0.5802" "0.4911" "rs2272662" "0.281" "T" "0.139" "B" "0.088" "B" "0.173" "N" "0.999" "P" "0.86" "L" "0.88" "T" "-0.73" "N" "0.069" "-0.454" "0.285" "0.743" "0.306" "N" "-0.943" "T" "0.000" "T" "0.403" "0" "-0.978" "-0.291" "-0.350" "0.546" "0.097" "3.810" 1 275.78 9 "chr8" 145639726 "." "T" "C" 275.78 "." "AC=2;AF=1.00;AN=2;DP=9;FS=0.000;MLEAC=2;MLEAF=1.00;MQ=60.00;MQ0=0;QD=30.64" "GT:AD:DP:GQ:PL" "1/1:0,9:9:27:304,27,0"

"24" "chr8" 145640411 145640411 "A" "G" "exonic" "SLC39A4" "." "nonsynonymous SNV" "SLC39A4:NM_017767:exon3:c.T676C:p.W226R;SLC39A4:NM_130849:exon4:c.T751C:p.W251R" "8q24.3" "0.9678" "0.9933" "0.9868" "0.9997" "0.9482" "0.9536" "0.9754" "0.9862" "rs2977838" "." "." "." "." "." "." "." "." "." "." "." "." "." "." "." "." "." "." "." "." "." "." "." "." "." "." "." "." "." "." "." "." "." "." 1 196.8 7 "chr8" 145640411 "." "A" "G" 196.8 "." "AC=2;AF=1.00;AN=2;DP=7;FS=0.000;MLEAC=2;MLEAF=1.00;MQ=56.62;MQ0=0;QD=28.11" "GT:AD:DP:GQ:PL" "1/1:0,7:7:21:225,21,0"

"25" "chr8" 145641328 145641328 "C" "T" "exonic" "SLC39A4" "." "nonsynonymous SNV" "SLC39A4:NM_017767:exon1:c.G265A:p.A89T;SLC39A4:NM_130849:exon2:c.G340A:p.A114T" "8q24.3" "0.4975" "0.3296" "0.4403" "0.4125" "0.5816" "0.5572" "0.4791" "0.4136" "rs17855765" "1.0" "T" "0.804" "P" "0.124" "B" "0.683" "N" "1" "P" "0.345" "N" "0.43" "T" "-0.17" "N" "0.05" "-0.115" "1.618" "0.933" "0.032" "N" "-0.940" "T" "0.000" "T" "0.646" "0" "-3.67" "-0.392" "-0.659" "0.001" "0.001" "10.636" 1 37.74 2 "chr8" 145641328 "." "C" "T" 37.74 "." "AC=2;AF=1.00;AN=2;DP=2;FS=0.000;MLEAC=2;MLEAF=1.00;MQ=60.00;MQ0=0;QD=18.87" "GT:AD:DP:GQ:PL" "1/1:0,2:2:6:65,6,0"

"26" "chr8" 145641417 145641417 "G" "A" "exonic" "SLC39A4" "." "nonsynonymous SNV" "SLC39A4:NM_017767:exon1:c.C176T:p.P59L;SLC39A4:NM_130849:exon2:c.C251T:p.P84L" "8q24.3" "0.0380" "0.0053" "0.0294" "0" "0.1127" "0.0533" "0.0344" "0.0112" "rs117535951" "0.143" "T" "0.645" "P" "0.113" "B" "0.074" "N" "1" "N" "0.345" "N" "0.55" "T" "-1.44" "N" "0.04" "0.739" "9.082" "0.963" "0.088" "N" "-1.091" "T" "0.018" "T" "0.581" "0" "-1.05" "-0.424" "0.059" "0.000" "0.000" "3.500" 1 188.81 7 "chr8" 145641417 "." "G" "A" 188.81 "." "AC=2;AF=1.00;AN=2;DP=8;FS=0.000;MLEAC=2;MLEAF=1.00;MQ=60.00;MQ0=0;QD=23.60" "GT:AD:DP:GQ:PL" "1/1:0,7:7:20:217,20,0"

"27" "chr8" 145641564 145641564 "T" "G" "exonic" "SLC39A4" "." "nonsynonymous SNV" "SLC39A4:NM_017767:exon1:c.A29C:p.E10A" "8q24.3" "0.5157" "0.4817" "0.4476" "0.4045" "0.5750" "0.5588" "0.5204" "0.4398" "rs2280839" "0.0" "D" "0.0" "B" "0.001" "B" "." "." "1" "P" "0" "N" "0.09" "T" "-0.18" "N" "0.071" "0.299" "5.683" "0.950" "0.029" "N" "-0.926" "T" "0.000" "T" "0.554" "0" "-3.1" "-0.221" "-0.071" "0.000" "0.004" "5.3" 1 344.77 11 "chr8" 145641564 "." "T" "G" 344.77 "." "AC=2;AF=1.00;AN=2;DP=11;FS=0.000;MLEAC=2;MLEAF=1.00;MQ=57.87;MQ0=0;QD=31.34" "GT:AD:DP:GQ:PL" "1/1:0,11:11:33:373,33,0"

"28" "chr8" 145642002 145642002 "C" "T" "exonic" "SLC39A4" "." "nonsynonymous SNV" "SLC39A4:NM_130849:exon1:c.G172A:p.A58T" "8q24.3" "0.5757" "0.4881" "0.5190" "0.4549" "0.6372" "0.6259" "0.5696" "0.5044" "rs2280838" "0.976" "T" "0.0" "B" "0.0" "B" "0.100" "N" "1" "P" "0.345" "N" "0.38" "T" "0.24" "N" "0.385" "0.573" "7.970" "0.945" "0.104" "N" "-1.026" "T" "0.000" "T" "0.701" "0" "-9.42" "-0.706" "-1.955" "0.965" "0.341" "5.707" 1 147.9 5 "chr8" 145642002 "." "C" "T" 147.9 "." "AC=2;AF=1.00;AN=2;DP=6;FS=0.000;MLEAC=2;MLEAF=1.00;MQ=56.82;MQ0=0;QD=24.65" "GT:AD:DP:GQ:PL" "1/1:0,5:5:15:176,15,0"

"29" "chr8" 145737636 145737636 "A" "G" "exonic" "RECQL4" "." "synonymous SNV" "RECQL4:NM_004260:exon19:c.T3127C:p.L1043L" "8q24.3" "0.9728" "0.9942" "0.8977" "0.7806" "0.9973" "0.9991" "0.9828" "0.9984" "rs4925828" "." "." "." "." "." "." "." "." "." "." "." "." "." "." "." "." "." "." "." "." "." "." "." "." "." "." "." "." "." "." "." "." "." "." 1 484.77 14 "chr8" 145737636 "." "A" "G" 484.77 "." "AC=2;AF=1.00;AN=2;DP=14;FS=0.000;MLEAC=2;MLEAF=1.00;MQ=57.28;MQ0=0;QD=34.63" "GT:AD:DP:GQ:PL" "1/1:0,14:14:42:513,42,0"

"30" "chr8" 145740364 145740364 "G" "A" "exonic" "RECQL4" "." "nonsynonymous SNV" "RECQL4:NM_004260:exon9:c.C1576T:p.L526F" "8q24.3" "0.0007" "0" "0.0007" "0" "0" "0.0008" "0.0014" "0.0009" "rs200732690" "." "." "0.382" "B" "0.519" "P" "." "." "." "." "." "." "." "." "." "." "0.37" "2.025" "16.37" "0.871" "0.849" "D" "." "." "." "." "0.284" "0" "3.49" "-0.045" "0.045" "0.654" "0.897" "7.169" 1 146.9 5 "chr8" 145740364 "." "G" "A" 146.9 "." "AC=2;AF=1.00;AN=2;DP=5;FS=0.000;MLEAC=2;MLEAF=1.00;MQ=55.21;MQ0=0;QD=29.38" "GT:AD:DP:GQ:PL" "1/1:0,5:5:15:175,15,0"

"31" "chr8" 145742514 145742514 "A" "G" "exonic" "RECQL4" "." "nonsynonymous SNV" "RECQL4:NM_004260:exon4:c.T274C:p.S92P" "8q24.3" "0.9733" "0.9799" "0.8983" "0.8026" "0.9972" "0.9991" "0.9837" "0.9984" "rs2721190" "." "." "." "." "." "." "." "." "." "." "." "." "." "." "." "." "0.043" "-1.106" "0.010" "0.453" "0.001" "N" "." "." "." "." "0.207" "0" "1.99" "-2.037" "-0.881" "0.000" "0.000" "5.147" 1 352.77 12 "chr8" 145742514 "." "A" "G" 352.77 "." "AC=2;AF=1.00;AN=2;DP=12;FS=0.000;MLEAC=2;MLEAF=1.00;MQ=60.00;MQ0=0;QD=29.40" "GT:AD:DP:GQ:PL" "1/1:0,12:12:35:381,35,0"

"32" "chr19" 3546340 3546340 "G" "A" "exonic" "MFSD12" "." "synonymous SNV" "MFSD12:NM_001287529:exon7:c.C1080T:p.Y360Y;MFSD12:NM_174983:exon7:c.C1107T:p.Y369Y" "19p13.3" "0.2010" "0.3695" "0.1673" "0.0118" "0.2506" "0.2003" "0.1769" "0.2085" "rs11555328" "." "." "." "." "." "." "." "." "." "." "." "." "." "." "." "." "." "." "." "." "." "." "." "." "." "." "." "." "." "." "." "." "." "." 0.5 75.77 5 "chr19" 3546340 "." "G" "A" 75.77 "." "AC=1;AF=0.500;AN=2;BaseQRankSum=1.231;ClippingRankSum=-1.231;DP=5;FS=3.979;MLEAC=1;MLEAF=0.500;MQ=60.00;MQ0=0;MQRankSum=1.231;QD=15.15;ReadPosRankSum=0.358" "GT:AD:DP:GQ:PL" "0/1:2,3:5:55:104,0,55"
